# In Vitro, Anti‐Colon Cancer Activity of Green Dumbbell‐Shaped *Rhododendron luteum*‐Based Carbon Dots

**DOI:** 10.1002/open.202400303

**Published:** 2025-01-29

**Authors:** Alper Durmaz, İbrahim Mizan Kahyaoğlu, Erdi Can Aytar, Ferdane Danışman Kalındemirtaş, Esra Sert, Ayşe Erol‐Bozkurt, Selcan Karakuş

**Affiliations:** ^1^ Ali Nihat Gökyiğit Botanical Garden Application and Research Center Artvin Çoruh University 08000 Artvin Türkiye; ^2^ Department of Chemistry Faculty of Science Ondokuz Mayıs University 55139 Samsun Türkiye; ^3^ Department of Horticulture Faculty of Agriculture Usak University 64200 Usak Türkiye; ^4^ Department of Physiology Faculty of Medicine Erzincan Binali Yildirim University 24100 Erzincan Türkiye; ^5^ Department of Hematology Istanbul Faculty of Medicine Istanbul University 34093 Istanbul Türkiye; ^6^ Department of Medical Biology Istanbul Faculty of Medicine Istanbul University 34093 Istanbul Türkiye; ^7^ Department of Chemistry Faculty of Engineering Istanbul University-Cerrahpaşa 34320 Istanbul Türkiye; ^8^ Health Biotechnology Joint Research and Application Center of Excellence, Esenler 34220 Istanbul Türkiye

**Keywords:** Anticancer, Green nanostructure, HCT116 colon cancer cell, MCF-7 breast cancer, *Rhododendron luteum*

## Abstract

Colorectal cancer is the second most common cause of cancer‐related deaths worldwide and the third most common cancer overall. In this study, we investigate the anti‐colon cancer potential of phytochemically, and thermally synthesised novel green carbon dots based on *Rhododendron luteum* (RL‐CDs). A new synthesis method was used to produce carbon dots obtained from the *Rhododendron luteum* (RL) plant in an environmentally friendly manner. The green RL were characterized using Fourier‐transform infrared spectroscopy (FTIR), UV‐Vis spectroscopy, transmission electron microscopy (TEM), and artificial intelligence (AI)‐based TEM analysis. The FTIR spectrum showed peaks corresponding to the hydroxyl (−OH) vibration of polyphenols at 3500 cm^−1^, the C=O vibration of cellulose derivatives at 1728 cm^−1^, and the C−O stretching of primary alcohol at 1041 cm^−1^. Two UV absorption peaks at roughly 253 nm (UV−C range), and 320 nm (UV−B range) were observed. The size of the green RL was measured to be less than 50 nm, and its morphology was characterized as dumbbell‐shaped through TEM analysis. In‐vitro studies were performed with HCT116 colon cancer, MCF‐7 breast cancer, and normal HUVEC cells. The results demonstrated that the RL‐CDs exhibited selective cytotoxic activity against HCT116 colon cancer cells. The results show that the RL extract stimulates cancer cell death by decreasing the CD44/24 ratio, and increasing apoptotic activity. These observations suggest that green RL‐CDs could be an effective anticancer agent in colon cancer therapy, investigating their potential in this direction could be a promising way for future research.

## Graphical abstract

The anticancer potential of *Rhododendron luteum*‐based carbon dots was investigated. Environmentally friendly synthesis yielded carbon dots that selectively induced cytotoxicity in HCT116 cells by reducing CD44/24 ratio, causing G2/M phase arrest, and enhancing apoptosis. These findings suggest their promising application in colon cancer therapeutics.

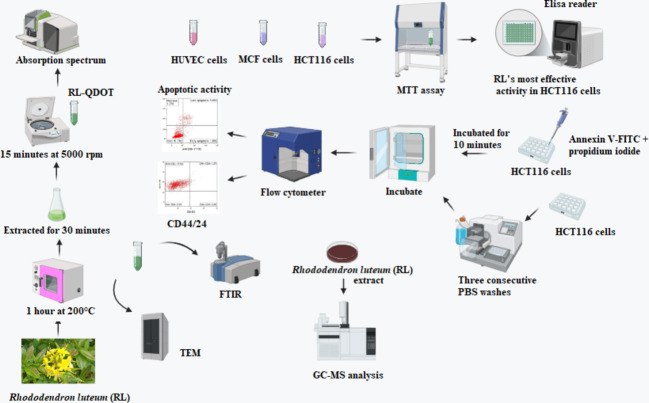

## 1 Introduction

The genus *Rhododendron* L. comprises around 850 species distributed globally within the Ericaceae family. Numerous secondary metabolites produced by these plants are beneficial in the management of chronic inflammation and have low toxicity.[^1^, ^2^] Polyphenols, specifically flavonoids and terpenes, are the primary phytochemicals found in species belonging to the genus *Rhododendron*, commonly referred to as rhododendrons. *Rhododendron luteum* Sweet in Hort. Brit., ed. 2 : 343 (1830) is a species characteristic of this genus. It is sometimes referred to as yellow azalea or honeysuckle azalea. *R. luteum* possesses several intriguing properties, such as suppressing bacterial growth and exhibiting cytotoxicity. Furthermore, it has been demonstrated that *R. luteum* leaf extracts can inhibit tyrosinase, α‐glucosidase, butyrylcholinesterase, and acetylcholinesterase.^[3]^ Numerous free and bound phenolic compounds, including as caffeic acid, p‐coumaric acid, protocatechuic acid, myricetin, and quercetin, are found in *R. luteum* samples. Examples of these compounds include 5‐O‐caffeoylquinic acid, ferulic acid, protocatechuic acid, catechin, and dihydromyricetin. High potential anti‐inflammatory *R. luteum* samples can inhibit hyaluronidase and lipoxygenase. There are certain samples that can block both cyclooxygenase 1 and cyclooxygenase 2. It possesses moderate Fe^2+^ ion chelating capabilities, radical scavenging ability, and antioxidant activity.^[1]^ Numerous secondary metabolites, including alkaloids, flavonoids, glycosides, saponins, tannins, steroids, and phlobatannins, are found in *Rhododendron* species and are vital to human health. They also possess a wide range of biological and medicinal actions, including insecticidal, antipyretic, antiviral, anti‐inflammatory, anti‐nociceptive, and anti‐hypertensive qualities.^[4]^

In previous studies, Turan et al. (2021) demonstrated that *R. luteum* has a specific lethal effect on HeLa cells, causing the cell cycle to halt in the S phase and triggering apoptosis, ER stress, and the production of ROS. They showed that in HeLa cells, *R. luteum* markedly increased CHOP expression while suppressing the expression levels of Nrf2, GCLC, and G6PD. These findings indicate that the Nrf2 and ER stress pathways are involved in the antiproliferative action of *R. luteum*.^[5]^ According to Mahomoodally et al. (2020), the aqueous extract of *R. luteum* had an IC_50_ value of 207.2 μg/mL, indicating its ability to inhibit the growth of A549 cells. However, this extract has not received much scientific attention due to the presence of grayanotoxins. In terms of free radical scavenging, the methanol and water extracts of *R. luteum* show the highest activity, while the ethyl acetate extract exhibits the greatest metal‐chelating capabilities. The IC_50_ value for enzyme inhibition of the *R. luteum* water extract was also found to be 207.2 μg/mL.^[3]^ In another study, the *R. luteum* (RL) leaf extract demonstrated specific cytotoxicity against colon (1.9 times) and liver (2.2 times) cancer cells compared to normal fibroblast cells, according to a 2018 study by Demir et al. However, this selective cytotoxicity was not observed in breast cancer cells.^[6]^

The distinctive characteristics and multiple applications of zero‐dimensional (0D) carbon nanomaterials, like carbon quantum dots and fullerenes, have shown tremendous potential in cancer therapy. These agents have several important advantages that allow them beneficial for the fight against cancer with advanced green chemistry approaches.[^7^, ^8^, ^9^, ^10^] Recent studies have emphasised the interesting anticancer effects of carbon quantum dots (CQDs), nanoscale carbon‐based materials with unique physicochemical features. Through complex cellular and molecular interactions, CQDs have been shown to cause apoptosis, limit proliferation, and selectively target specific regions. Their customisable shape, size, surface functionalisation abilities, structure, solubility, and biological properties render them ideal for cancer‐targeted drug delivery and imaging uses. Das et al. reported graphene quantum dots (GQDs) as a possible cancer therapy due to their biocompatibility and distinctive characteristics. Pristine and doped GQDs (N‐GQD, S‐GQD) showed synergistic effects with methotrexate, with pristine GQDs causing 94.4 % HeLa cell death and doped variations remaining biocompatible with healthy cells. This study demonstrates GQDs’ potential for tailored cancer treatment.^[11]^ Weng et al. showed that graphene quantum dots (GQDs), an emergent quasi‐zero‐dimensional nanostructure, excel in targeted drug delivery and controlled release systems. Molecular dynamics simulations demonstrated that, unlike single molecules such as doxorubicin (DOX) or deoxyadenine (DA), GQD7 can form a sandwich shape with DOX, allowing it to translocate into POPC lipid membranes without affecting barrier integrity. GQDs, on the other hand, interacted only minimally with DA. These findings shed light on the chemical structure of GQD‐based drug delivery systems, highlighting their potential for use in improved medical uses.^[12]^ In summary, CQDs are regarded as potential therapeutic agents in cancer therapy due to their stability, excitation‐dependent emission, and simplicity of surface modification. These achievements highlight the promise of CQDs as new cancer therapeutic agents, necessitating future research to fully exploit their potential.

The novelty of this study lies in the eco‐friendly synthesis of *Rhododendron luteum* extract‐based carbon dots (RL‐CDs) using a unique phytochemical and thermal method, offering an effective alternative therapy for colon cancer. For the first time, RLs were shown to have a distinct dumbbell‐shaped morphology with a size of less than 50 nm. In‐vitro experiments were performed with MCF‐7 breast cancer cells, HCT116 colon cancer cells, and normal HUVEC (human umbilical vein endothelial cells). The green RL demonstrated selective anticancer activity against HCT116 colon cancer cells by reducing the CD44/CD24 ratio, inducing cell cycle arrest in the G2/M phase, and promoting apoptosis while it showed minimal cytotoxicity on normal HUVEC cells. Remarkably, RL showed selective cytotoxicity against HCT116 colon cancer cells, further emphasising its potential as a sustainable and promising candidate for colon cancer therapeutics. These findings underscore the favorable safety profile of green RLs and their potential as a selective and effective anticancer treatment, particularly for colon cancer.

## Material and Method

### Cytotoxicity

The American Type Culture Collection (ATCC) provided HCT116 (colon cancer cell line), MCF‐7 (breast cancer cells), and HUVEC (human umbilical cord vascular endothelial cells) cells for this study. At 37 °C in a humidified incubator atmosphere of 95 % O_2_, 5 % CO_2_, these cells were cultured in DMEM containing 10 % high‐temperature inactivated FBS, prepared by adding 100,000 U/L penicillin and streptomycin. The cells were plated in a 96‐well flat‐bottom plate with 10^4^ cells per well, and the synthesized RL extract was added directly at a 1 : 1 ratio and diluted with medium at 1 : 2, 1 : 4, and 1 : 8 ratios, and these cells were added. It was incubated in a humidified oven atmosphere with 5 % CO_2_ for 48 hours at 37 °C. Therefore, 10 μL MTT (5 mg/mL/PBS) was added to each well after 48 hours of incubation and left for 4 hours. The formazan crystals formed in the wells were then dissolved with 100 μL DMSO, and the optical density was measured using an ELISA reader at 570 nm and 630 nm reference wavelengths.

### Apoptotic Activity

Because HCT116 cells are the most affected by RL, apoptotic activity was investigated in these cells. Following 48 hours of treatment at the RL's approximate IC_50_ value for HCT116 cells, 5 μL of Annexin V‐FITC and 2.5 μL of propidium‐iodide were added to cells that had been prepared for the Annexin V/PI method of assessing apoptosis. The cells were then incubated for 10 minutes in the dark. A Beckman Coulter Navios flow cytometer (Navios 3 L10) was used to evaluate the treated cells, and 400 microliters of annexin binding buffer were added. The rates of necrosis and early‐to‐late apoptosis in these cells were assessed using Beckman Coulter flow cytometry, and the data were examined using Kaluza software. Because necrotic cells have broken membranes that let Annexin V access the entire plasma membrane, Annexin V can also stain necrotic cells. Furthermore, co‐treatment with propidium iodide (PI) allows for the differentiation of apoptotic from necrotic cells. PI can enter necrotic cells while avoiding apoptotic ones.^[13]^

### CD44/24

After treating HCT116 cells for 48 hours at the approximate IC_50_ value of the RL extract, CD44, and CD24 ratios were prepared for flow cytometry after three consecutive PBS washes. Cells were stained with CD44 and CD24 and incubated with these antibodies for 15–20 minutes at room temperature and in the dark for this purpose. The HCT116 cells were then examined on the Beckman Coulter Navios device using flow cytometry and the Kaluza analysis program. Between ideal wavelengths, compatibility (488–561 nm) and emission (578 nm) were discovered. Each tube had a minimum of 10,000–15,000 cells read.

### Collecting and Drying Plants

The plant materials of *Rhododendron luteum* were collected from Mahmutlu village in the Kavak district of Samsun province and from the edges of Carpinus forests in the Yeşilırmak delta, where the populations are dense and uniform. Dr. Alper Durmaz identified the species.

These plant samples are stored under accession number OMUB‐8080 in the Herbarium of the Biology Department at Ondokuz Mayıs University. The collected materials were dried in ovens at 50 degrees Celsius for 72 hours in the soil and plant laboratory. After drying, the *Rhododendron luteum* samples were ground into a powder using a blender. The powdered material is kept in airtight zip‐lock bags in a cool, dry place until analysis. Only the flowers of the *Rhododendron luteum* plant were collected for study. All analyses and research were carried out on the dried, ground flower material.

### Thermal Synthesis of Green RL

The pyrolysis method was selected for the synthesis of green RL. To carry out this process, 2 g of the sample were heated at 200 °C for 1 hour. Subsequently, 20 mL of distilled water was added to the sample, and the green RL formed were extracted for 30 minutes. The extraction solution was then centrifuged at 5000 rpm for 15 minutes. The supernatant was diluted with distilled water at a 1 : 20 ratio, and absorption spectra in the range of 200–1000 nm were recorded using quartz cuvettes, with distilled water serving as the blank.

### Characterization

The prepared green nanostructure was characterized using different techniques such as TEM (Hitachi HighTech HT7700 model, in a high vacuum mode at 100 Kv), UV‐Vis spectroscopy (PG INSTRUMENTS‐T80+UV‐VIS double beam model), and FTIR (Perkin Elmer, Spectrum Two model) in the 4000–400 cm^−1^ frequency range with a resolution of 4 cm^−1^ and 8 scans.

### GC‐MS Analysis

GC‐MS analysis was performed using an Agilent gas chromatograph (Agilent Technologies, Santa Clara, CA, USA) equipped with an HP‐88 capillary column (60 m×0.25 mm×0.20 μm). High‐purity helium (>99.99 %) was employed as the carrier gas at a constant flow rate of 1.0 mL/min. The injection volume was 1 μL, with a split ratio of 1 : 50, and the injector temperature was maintained at 250 °C. The oven temperature program was set to an initial temperature of 140 °C, held constant for 5 minutes, followed by an increase at a rate of 4 °C/min to reach 250 °C, which was maintained for 10 minutes. The total GC‐MS run time was approximately 48 minutes. Mass spectrometric analysis was conducted using electron ionization at 70 eV. The ion source temperature was set to 230 °C, and the mass scanning range was configured between m/z 30 and 550. Samples were analyzed in a 1.5 mL vial after preparation. All compounds were identified using the NIST Mass Spectral Database.

## 2 Results and Discussion

In the present study, we conducted a GC‐MS analysis to identify the major phytochemical components of the flower extract of *Rhododendron luteum*. This analysis provides essential insights into the chemical composition of the extract, laying a foundation for understanding its potential biological activities.

The GC‐MS analysis revealed several prominent compounds in the flower extract, with Morphine, 2TMS derivative being the most abundant at 13.012 %, followed by Pentane, 2,2,3,4‐tetramethyl‐ at 12.849 %. Furthermore, 9,12,15‐Octadecatrienoic acid, methyl ester, (Z,Z,Z)‐ (11.956 %) and Propanoic acid, 2‐methyl‐ (9.319 %) were identified as key constituents. Noteworthy additional compounds included benzaldehyde (1.098 %) and galangin (3.932 %), both recognized for their potential biological significance (Table 1). These results underscore the chemical diversity of the *Rhododendron luteum* flower extract and its promising implications for future biological and pharmacological investigations.

**Table 1:** Major phytochemical components identified in the flower extract of *Rhododendron luteum* through GC‐MS analysis.

No	RT (min)	Name of the Compound	Content [%]
1	2.484	2‐Propanone	0.371
2	2.575	Acetone	0.673
3	2.768	Pentane, 2‐methyl‐	0.426
4	2.859	Furan, tetrahydro‐2‐methyl‐	1.619
5	2.95	Pentane, 3‐methyl‐	5.683
6	3.041	Pentane, 2,2,3,4‐tetramethyl‐	12.849
7	3.148	Propanoic acid, 2‐methyl‐	9.319
8	12.385	Benzaldehyde	1.098
9	13.315	1‐Octen‐3‐ol	2.553
10	13.995	(2R,5R)‐2‐Methyl‐5‐(prop‐1‐en‐2‐yl)‐ 2‐vinyltetrahydrofuran	2.213
11	14.76	Cyclopentane, ethylidene‐	1.678
12	15.615	o‐Cymene	0.983
13	15.829	D‐Limonene	0.755
14	18.204	alpha‐Terpineol	0.886
15	24.318	Dill ether	2.182
16	41.407	trans‐Calamenene	0.531
17	48.499	3,4‐Dihydroxymandelic acid	5.471
18	48.569	3,4‐Dihydroxyphenylglycol	6.123
19	54.319	Morphine, 2TMS derivative	13.012
20	55.394	Adenosine, 2‐methyl‐	0.758
21	56.367	Hexadecanoic acid, methyl ester	2.425
22	57.565	2,4‐Dihydroxyacetophenone, 2TMS derivative	4.754
23	59.395	9,12‐Octadecadienoic acid (Z,Z)‐, methyl ester	1.797
24	59.496	9,12,15‐Octadecatrienoic acid, methyl ester, (Z,Z,Z)‐	11.956
25	59.892	Methyl stearate	0.666
26	60.074	3,4‐Dihydroxymandelic acid, 4TMS derivative	2.492
27	61.689	Galangin	3.932
28	62.775	Communic Acid, TMS Derivative	2.795

In this study, the surface and chemical properties of the prepared green RL were characterized using TEM, UV, and FTIR analysis. TEM is a widely used technique for nanostructure characterization, allowing detailed visualization of small particles. However, the interpretation of TEM images poses challenges due to the small particle sizes, selection bias, and the complexity of quantifying intricate features. To address these issues, an AI‐based method was employed to extract information from TEM results with minimal human intervention. Specifically, AI‐supported FIJI software was utilized for advanced image processing, which enabled the generation of pixel intensity histograms to assess particle size and distribution. Additionally, the software facilitated the enhancement of TEM images in both 32‐bit RGB and 8‐bit formats, providing clearer visualization of structural details. This approach improved accuracy, minimized bias, and ensured a more robust analysis of the nanostructures.

**Figure 1:**
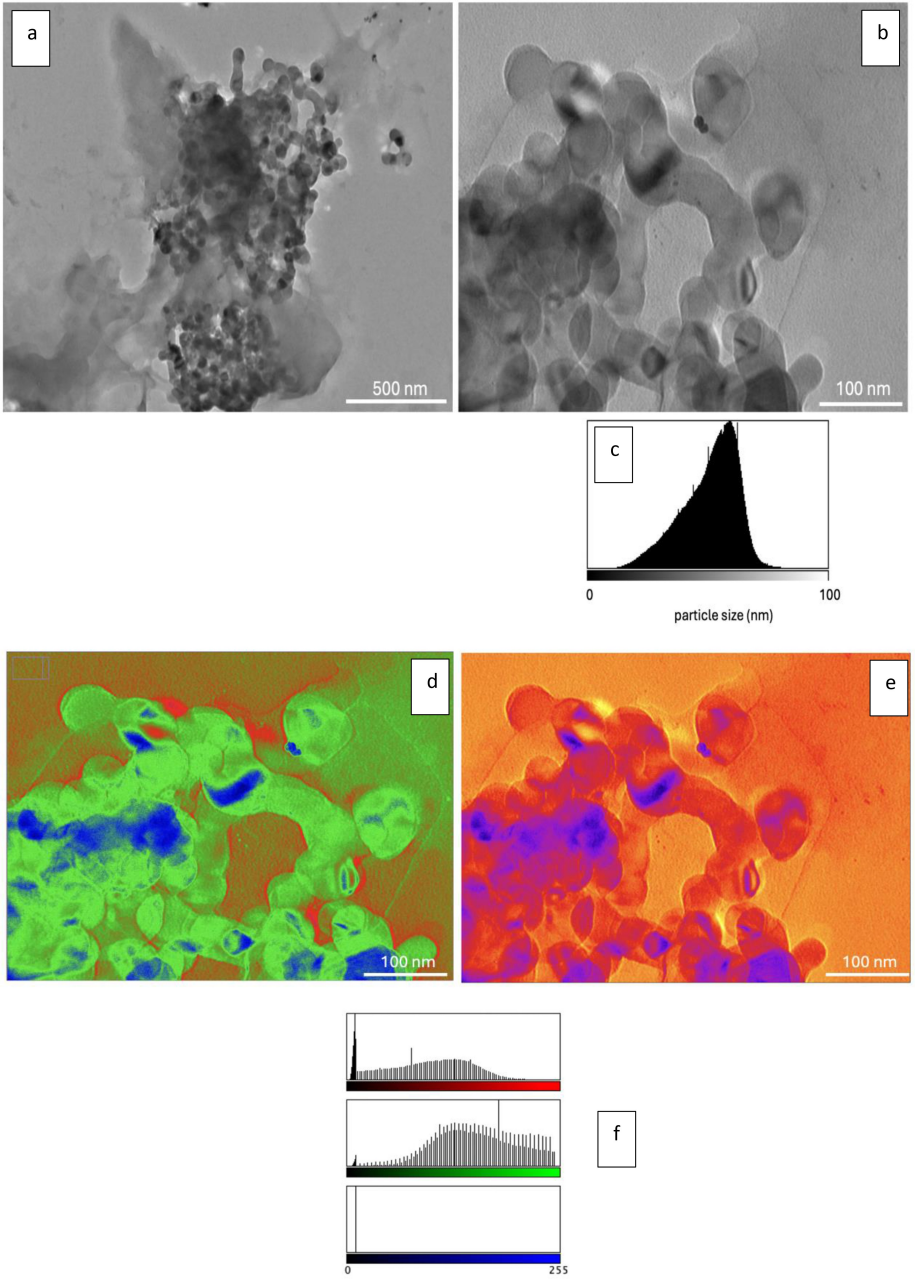
TEM micrographs of RL at a) 15.0 k magnification and b) 60.0 k magnification, c) TEM histogram of RL, d) 32‐bit RGB color‐enhanced TEM image, e) 8‐bit pixel color‐enhanced TEM image, and f) color histogram.

In this study, the histogram of pixel intensity from AI‐assisted TEM images of the RL was utilized to assess its size, dispersion, and stability. In Figure 1, TEM micrographs of RL at a) 15.0 k magnification and b) 60.0 k magnification, c) TEM histogram of RL, d) 32‐bit Red‐Green‐Blue (RGB) mode color‐enhanced TEM image, e) 8‐bit pixel color‐enhanced TEM image, and f) color TEM histogram were presented. In TEM micrographs of RL taken at both 15.0 k and 60.0 k magnification, the nanoscale structure is clearly visible. To perform a clearer surface analysis, AI‐supported FIJI software was used for image processing. Consequently, 32‐bit and 8‐bit color‐enhanced TEM images were obtained. The TEM images showed that the particles were in partial aggregates, which is a common occurrence in nanostructure characterization due to the high surface energy of nanostructures causing them to cluster. Despite this aggregation, the experimental results clearly demonstrated that the individual size of the RL was less than 50 nm. Detailed TEM analysis further revealed their distinct dumbbell‐shaped morphology, highlighting the unique structural characteristics of these CQDs. The observed aggregation did not impact the determination of individual particle size or morphology, which was accurately assessed using AI‐supported image processing for enhanced visualization and precise measurement.

**Figure 2:**
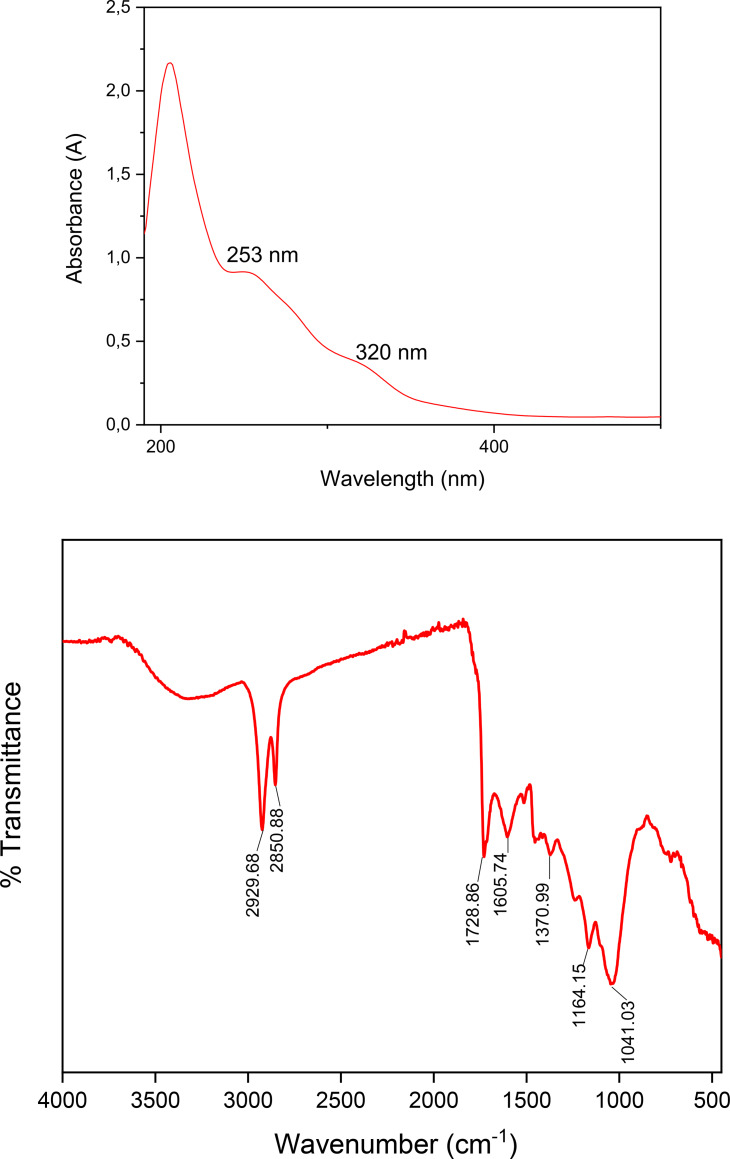
a) UV‐Vis spectra and b) FTIR graph of RL.

The hydrophilicity and enhanced capacity for functionalization with diverse organic, biological, and polymeric molecules are attributed to the functional groups present on the surface of carbon dots (CDs). Due to the π‐π* transitions of C=C bonds, CDs actively gather photons in the short wavelength region. The UV region is divided into three bands spanning the wavelength range of 100–400 nm: UVA (320–400 nm), UVB (280–320 nm), and UVC (200–280 nm).^[14]^ CDs also exhibit significant absorption in the UV (260–320 nm) area, with a tail extending into the visible spectrum. Furthermore, the absorption wavelength of CDs is influenced by distinct surface functional groups. As seen in Figure 2, two UV absorption maxima for the RL were observed in this study at approximately 253 nm (optical band gap: 4.9 eV) in the UV−C range and 320 nm (optical band gap: 3.9 eV) in the UV−B range.^[15]^ It was observed that absorption increases as the wavelength decreases from longer to shorter wavelengths. As illustrated in Figure 2, there is an exponential rise in absorption as the wavelength transitions from 400 nm–310 nm, aligning with the UVA range.

The UV‐Vis absorption spectra of RL are displayed in Figure 2a; at 253 nm and 320 nm, there are two separate absorption peaks. The π‐π✶ transition of the C=C bond of the benzene ring linked to the carbon core surface is responsible for the absorption peak at 253 nm,^[16]^ whereas the n‐π✶ transition of the C=O bond is responsible for the absorption peak at 320 nm. Thus, the observed variances in their UV‐Vis spectra can be explained by variations in the electronic structures and compositions of RL.^[17]^

According to the FTIR results of RL (Figure 2b), the peak at 2929 cm^−1^ corresponds to the hydroxyl (−OH) vibration of polyphenols^[18]^ and the peak at 2850 cm^−1^ corresponds to the C−H vibration of cellulose and lipids.^[19]^ The peak at 1728 cm^−1^ is attributed to the C=O vibrations of cellulose derivatives,^[20]^ while the amide I vibrations of proteins are observed around 1605 cm^−1^.^[21]^ The asymmetric CH_2_ vibration of cellulose and lignin appears around 1370 cm^−1^.^[22]^ The peak at 1164 cm^−1^ corresponds to the C−O−C glycosidic bond,^[23]^ and the peak at 1041 cm^−1^ is attributed to the C−H vibration of lipids.^[24]^

**Figure 3:**
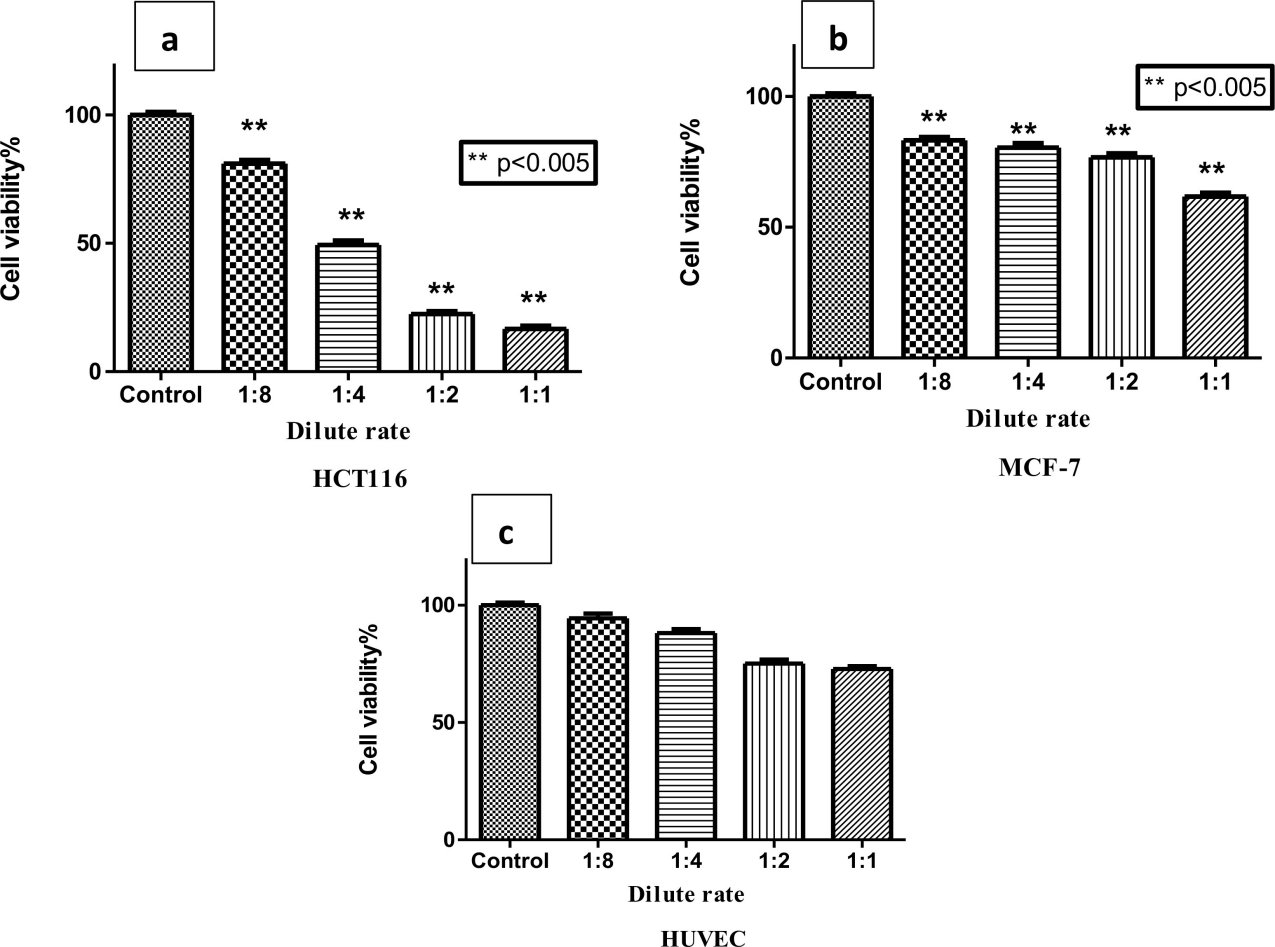
Cytotoxicity was evaluated by applying RL‐CDs at the concentrations shown in the graph. By diluting 1/8, 1/4, 1/2, and 1 on HCT116 (a), MCF‐7 (b), and HUVEC (b) cells, varying concentrations of RL‐CDs were applied. Statistical significance is demonstrated by p 0.005 : ** when compared to the control group. The student‐t test was used to analyze the samples. For statistical analysis, GraphPad Prism 8.0 (GraphPad Software, San Diego, CA) was utilized. At least three replications of each test were conducted.

It is noteworthy that the RL‐CDs have a specific effect on HCT116 colon cancer cells among the cell lines we examined. While the RL‐CDs killed about half of the HCT116 cells at a dilution of 1/4, they showed less cytotoxicity on MCF‐7 breast cancer cells than on HCT116; when the RL‐CDs were applied directly without dilution, about 40 % of the MCF‐7 cells died, while 60 % remained alive. It is noteworthy that the dilution ratio of RL‐CDs on HCT116 cells of 1 : 4 corresponds approximately to the IC_50_ value, which indicates the concentration at which 50 % of cell viability is inhibited. However, when considering the effect of the RL‐CDs on normal HUVEC cells, their cytotoxicity appears to be quite low. It is noteworthy that even when the RL‐CDs were applied directly (1/1) to HUVEC cells, 72 % of the HUVEC cells were still alive and that the cytotoxicity was much lower when diluted (Figure 3).

Programmed cell death, or apoptosis, is a necessary physiological process that needs to take place in order to eliminate damaged or undesirable cells. Apoptotic problems lead to cancer development and increase treatment resistance in cancerous cells.^[25]^

**Figure 4:**
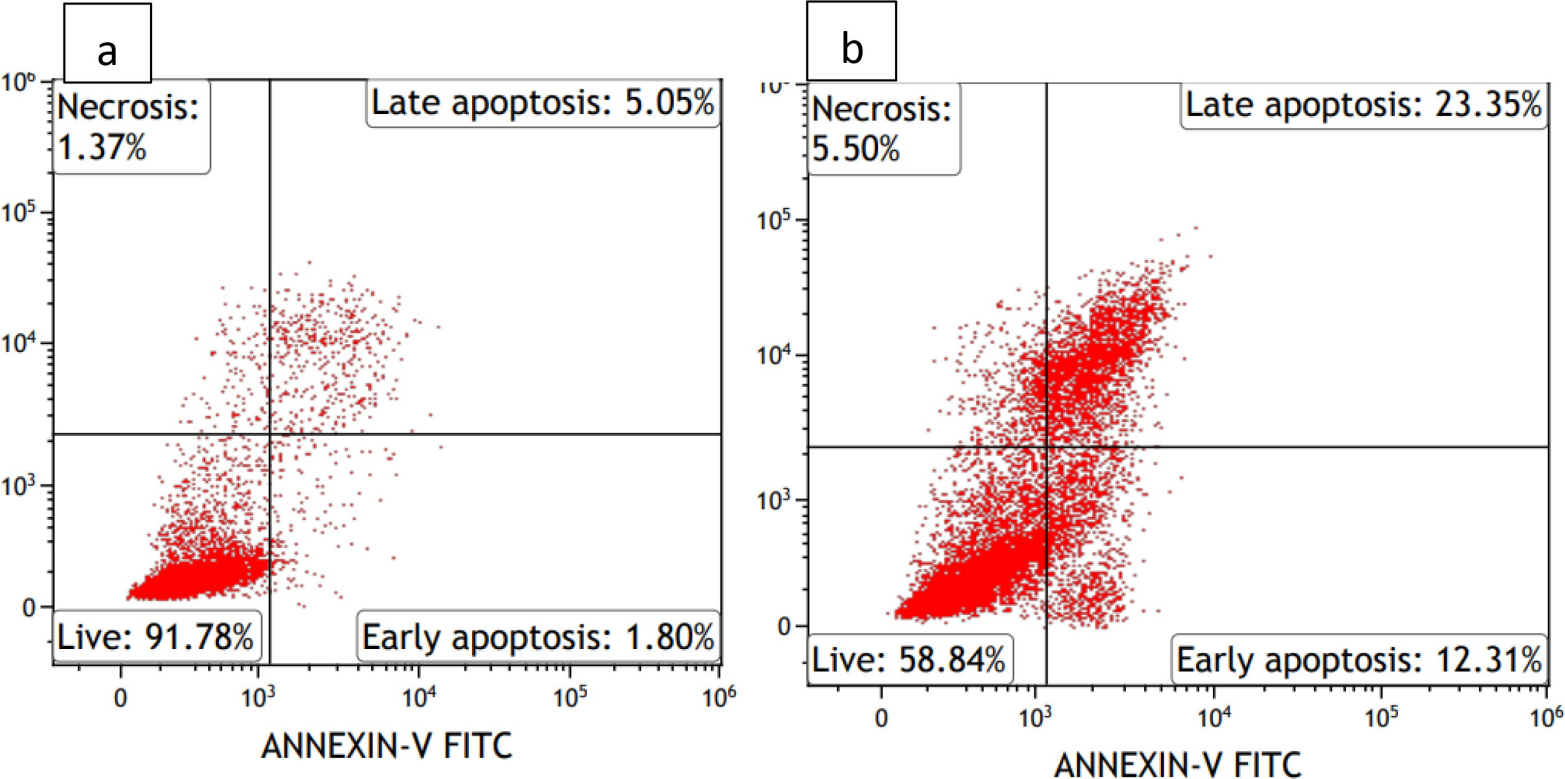
The apoptotic effects of RL‐CDs on HCT116 cells are depicted in Figure 2. HCT116 cells treated with RL‐CDs at nearly IC_50_ values (b) and untreated HCT116 cells (a).

When untreated HCT116 cells were stained with Annexin V and PI, 91.78 % of the cells were found to be live, 1.8 % were in early apoptosis, 5.05 % in late apoptosis and 1.37 % in necrosis. When HCT116 cells were treated with RL‐CDs at around IC_50_, the number of live cells decreased to 58.88 %, 12.31 % of cells underwent early apoptosis, 23.35 % late apoptosis and 5.5 % underwent necrosis. This indicated that the RL‐CDs caused cell death mainly by apoptosis (Figure 4).

The principal CD44 binding molecule, hyaluronic acid, has proven to be a valuable ally in the development of nanocarriers that exhibit preferential tumor accumulation and enhanced cell uptake.^[26]^ On cancer stem cells, the cell surface adhesion molecule CD44 is overexpressed. The growth and spread of tumors as well as the expression of the chemoresistant phenotype are caused by the interaction of CD44 with hyaluronan. The cytotoxic action of chemotherapy drugs is impeded in a variety of malignancies by the overexpression of CD44. Therefore, in those who are affected, a high expression of CD44 is linked to a bad prognosis.^[27]^ CD44 is widely employed as a surface marker for identifying CSCs (Cancer stem cells) in breast, prostate, pancreatic, ovarian, and colorectal malignancies.[^28^, ^29^] According to Ju et al., expression of CD24 reduces c‐Raf/mitogen‐activated protein kinase (MEK)/mitogen‐activated protein kinase signaling and cell proliferation. Furthermore, it was discovered in the same study that CD24 expression increased DNA‐induced apoptosis by inhibiting the antiapoptotic NF−B signal in CD44‐expressing cells.^[30]^

In our study, RL‐CDs were found to enhance apoptotic activity while reducing the CD44/CD24 ratio. These findings suggest that RL‐CDs hold promise for future research in the treatment of colon cancer.

The population of CD44+CD24 HCT116 cells is affected by RL‐CDs. Using flow cytometry, the proportion of cancer cells (CD44+/CD24−) in the HCT116 cell population was identified. Using flow cytometry, the expression patterns of CD24 and CD44 in HCT116 cells were examined.

**Figure 5:**
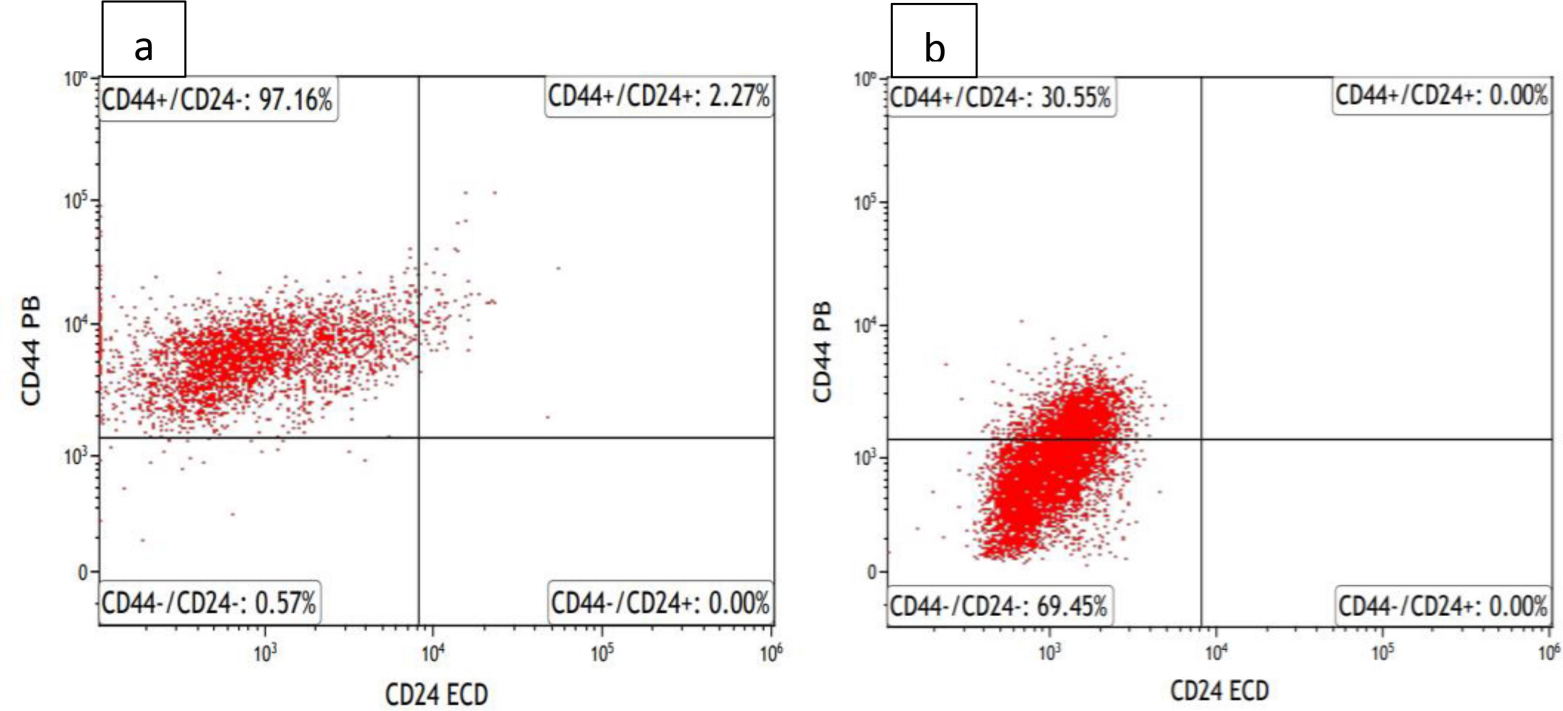
Analysis of the HCT116 cell surface marker, CD44+/CD24−, using flow cytometry. The percentage of CD44+/CD24− cell surface markers treated with RL extract at IC_50_ value is shown on the graph. Untreated HCT116 cells (a), HCT116 cells treated with RL (b).

While CD44+/CD24− expression was 97.16 % in untreated HCT116 cells, it was detected as 30.55 % in HCT116 cells treated with RL‐CDs (nearly IC_50_ value). Decreased expression of CD44, also known as a cancer marker; suggests that it is positively correlated with apoptosis and is an important marker due to its cytotoxicity‐enhancing effect. In this context, we observe that HCT116 cells treated with RL‐CDs exhibit no resistance to treatment, which is appropriately associated with cells undergoing apoptosis (Figure 5).

According to the literature, CQDs have selective anticancer activity through a variety of mechanisms.[^31^, ^32^, ^33^] CQDs exhibit enhanced uptake by cancer cells due to their higher endocytotic activity and rapid metabolism, particularly when functionalized with targeting ligands like folic acid or peptides. CQDs have increased absorption by tumor cells due to their stronger endocytotic activity and fast metabolic processes, especially when enhanced with targeted receptors such as folic acid/protein. They produce reactive oxygen species (ROS) and use the heightened oxidative stress in tumor cells to preferentially trigger apoptosis. Their activity in the acidic tumor milieu enables pH‐sensitive triggering or drug release, particularly in tumor tissue. CQDs can affect the membrane of the mitochondrial possibility, limiting ATP generation and inducing death, as cancer‐ridden mitochondria are especially vulnerable due to their hyperpolarization. Furthermore, CQDs can bind with DNA or proteins in cancerous cells, preventing replication and triggering an arrest of cell cycle. The modification improves the targeted effect by targeting cancer‐specific receptors like CD44, minimizing the impact on normal cells. These mechanisms, together with possible photodynamic and immune‐modulatory impacts, render CQDs an effective and targeted anticancer therapeutic strategy. Compared to other carbon nanomaterials, which demonstrated various cytotoxic effects on different cancer cell lines, RL specifically induced cell cycle arrest and apoptosis in HCT116 cells in this study (Table 2). Our findings suggest that the decrease in the CD44+/CD24− ratio in HCT116 cells treated with RL extract is important in terms of its effect on apoptosis and cytotoxicity.

**Table 2:** Comparison of the anticancer efficiency of various carbon nanomaterials.

Carbon nanomaterials	Characterizations	Biological Evaluations	Results	Ref
Chitosan/carbon quantum dots/Fe_2_O_3_ nanocomposite	Size: 227.2 nm	MTT assays showed cytotoxicity on MCF‐7 cells	Fe_2_O_3−_ loaded: Increases viability by 17 % compared to CS/CQDs, Curcumin‐loaded nanocarrier : Reduces cancer cell viability from 66 % (free curcumin)–48 %	[34]
Dacarbazine‐primed carbon quantum dots	Size: 2–10 nm	MDA‐MB‐231, MCF7 and 4 T1 cell lines	CQDs showed an IC_50_ value of 34.00 μM for MDA‐MB‐231 cells and 47.70 μM for MCF‐7 cells	[35]
Photo‐oxidase carbon dot‐based nanozyme	Size: 3–4 nm	B16‐F10 melanoma cells	the cell migration from 69 %–14 % within 24 h	[36]
Folic acid‐functionalized carbon dots	Size: 70 nm	MCF‐7 cells	the migration of MCF‐7 cells at a concentration of 50 μg/mL	[37]
Carbon quantum dots‐ quinic acid	Size: 7.55 nm	MCF‐7 cells	The IC_50_ values for Gemcitabine and N‐CQDs‐Quinic acid‐ Gemcitabine were 4.102 μg/ml and 2.908 μg/ml	[38]
Carbon dot anchored halloysite nanotube	Size: ∼50 nm	MCF‐7 cells	IC_50_ value was 84.85 %	[39]
Morus nigra‐derived hydrophilic carbon dots	Size: 4.5 nm	HTC‐116	No significant cytotoxicity was observed, with cell viability exceeding 97 % after 24 hours of incubation, even at a concentration of 200 mg/mL	[40]
Carbon‐based quantum dots	Size: 3.7 nm	mouse colon carcinoma (C26) and mouse bladder carcinoma (MBT‐2) cells	good cell viability towards two different cell lines	[41]
Copper‐doped saffron‐based carbon dots	Size: 20 nm	HCT‐116 and HT‐29 cells	HCT‐116: (IC_50_, 0.42 mg/ml), HT‐29: (IC_50_, 0.38 mg/ml)	[42]
Green RL	Size: <50 nm	Induced cell cycle arrest in the G2/M phase and supported the apoptosis in HCT116 cells	CD44+/CD24− expression was 97.16 % in untreated HCT116 cells, which decreased to 30.55 % in HCT116 cells treated with RL extract at the IC_50_ value	In this study

## 3 Conclusions

In this study, RL were successfully synthesized for the first time via a facile, simple, and thermal synthesis, introducing a new approach to green synthesis. Characterization techniques confirmed the formation and morphology of green RL as zero‐dimensional carbon nanomaterials (OD‐CMs) with a particle size of less than 50 nm, exhibiting a dumbbell shape. Our findings indicate that the RL‐CDs enhance apoptotic activity while reducing the CD44/24 ratio. In conclusion, the findings demonstrate that RL acts as a potent anticancer agent, effectively inducing apoptosis in HCT116 cells, offering promising potential for future therapeutic applications. The introduction of green RL‐based OD‐CMs marks a significant advancement in the field of nanomedicine and cancer treatment. The synthesis method offers a sustainable alternative to traditional carbon dot production, potentially reducing environmental impact. Furthermore, the selective anticancer activity of green RL opens new avenues for research in targeted cancer therapies, particularly for colon cancer. Future research should focus on exploring the detailed molecular mechanisms underlying the anticancer effects of RL‐based OD‐CMs and assessing their efficacy in vivo. By expanding the scope of green RL‐based OD‐CMs ′s applications, this study lays the groundwork for innovative and eco‐friendly solutions in cancer therapy.

## Author Contributions

AD: conceptualization; methodology; resources; writing–review and editing. AD, İMK, ECA, FDK, SK: validation; formal analysis; investigation; visualization; resources; writing–original draft. FDK, SK, ES, AB: formal analysis. SK: conceptualization; supervision; methodology; investigation; writing–review and editing. All authors have read and agreed to the published version of the manuscript.

## Conflict of Interests

The authors declare no conflict of interest.

4

## Data Availability

The data that support the findings of this study are available from the corresponding author upon reasonable request.
